# Pre-symptomatic scintigraphic and genetic cascade screening in cardiac transthyretin amyloidosis

**DOI:** 10.1007/s00259-024-06966-6

**Published:** 2024-11-14

**Authors:** Katarzyna Holcman, Paweł Rubiś, Bogdan Ćmiel, Agnieszka Stępień, Katarzyna Graczyk, Krystian Mróz, Wojciech Szot, Ewa Dziewięcka, Mateusz Winiarczyk, Maria Kurek, Mateusz Kęska, Piotr Podolec, Magdalena Kostkiewicz

**Affiliations:** 1 Jagiellonian University Medical College, Department of Cardiac and Vascular Diseases, Institute of Cardiology, St. John Paul II Hospital, Pradnicka 80, 31-202 Krakow, Poland; 2 Department of Nuclear Medicine, St. John Paul II Hospital, Krakow, Poland; 3 Faculty of Applied Mathematics, AGH University of Science and Technology, Krakow, Poland; 4 Doctoral School of Medical and Health Sciences, Jagiellonian University Medical College, Krakow, Poland; 5 Jagiellonian University Medical College, Department of Interventional Cardiology, Institute of Cardiology St, John Paul II Hospital, Krakow, Poland; 6 Department of Hygiene and Dietetics, Jagiellonian University Medical College, Krakow, Poland; 7 Students Scientific Group of Cardiovascular Imaging, Department of Cardiac and Vascular Diseases, Jagiellonian University Medical College, Krakow, Poland

**Keywords:** Transthyretin amyloidosis, ATTR, Transthyretin, DPD, SPECT, Amyloid cardiomyopathy

## Abstract

**Purpose:**

While early diagnosis is crucial, as new treatments can significantly slow the progression of the disease, there is growing evidence on the application of novel imaging techniques for detecting transthyretin amyloidosis (ATTR) in pre-symptomatic stages. This study aimed to evaluate the utility of pre-symptomatic scintigraphic imaging cascade screening for early detection of ATTR.

**Methods:**

During the period from 2020 to 2024, we conducted a prospective study that enrolled 100 consecutive adults. The study utilized a multimodal cascade screening approach to assess asymptomatic relatives of individuals with ATTR (ClinicalTrials.gov Identifier: NCT05814380). The analysis incorporated clinical data, genetic testing, echocardiography, scintigraphy and single-photon emission computed tomography/computed tomography (SPECT/CT) with [99mTc]Tc-DPD, regardless of the predicted age of disease onset.

**Results:**

Overall, scintigraphy identified cardiac amyloidosis (CA) in 8.2% of relatives, while 20.5% carried a pathogenic transthyretin variant without radiotracer uptake, with Phe53Leu being predominant. Notably, no relatives of wild-type ATTR patients exhibited CA on scintigraphy or carried a transthyretin variant. Additionally, newly-diagnosed relatives with ATTR CA presented elevated high-sensitivity troponin levels and exhibited a higher incidence of pathological electrocardiographic Q waves, greater thickness of the intraventricular septum and left ventricular posterior wall, a notable decline in lateral wall and intraventricular septal E' tissue velocities measured by TDI, and the "5–5-5" sign (*p* < 0.05).

**Conclusion:**

The presented findings demonstrate that implementing a systematic screening protocol, which integrates genetic and scintigraphic testing, facilitates the early detection of ATTR. Crucially, a significant proportion of asymptomatic relatives of patients with hereditary ATTR may suffer from underlying CA.

**Registration:**

ClinicalTrials.gov Identifier: NCT05814380.

## Introduction

Amyloid cardiomyopathy (CA) should be considered as one of the possible diagnoses in patients exhibiting increased left ventricular thickness or a clinical presentation consistent with restrictive cardiomyopathy [1, 2]. Transthyretin amyloidosis (ATTR) represents a progressive and often fatal disorder, challenging both in terms of initial diagnosis and subsequent treatment. This disease originates from the deposition of misfolded, insoluble transthyretin (TTR) fibrils in the extracellular matrix of the cardiac tissue and other organs. There are two distinct forms of ATTR: acquired wild-type ATTR (ATTRwt), and hereditary ATTR (ATTRv), inherited in an autosomal dominant pattern with variable expression of phenotypes among family members [3]. ATTR previously thought to be rare except in endemic regions, it is now diagnosed more frequently owing to heightened medical awareness and substantial advancements in non-invasive diagnostic techniques [4].

Scintigraphy using bone-avid tracers (3,3-disphono-1,2-propanodicarboxylic acid (DPD), methylenediphosphonicacid (MDP), and pyrophosphate (PYP)) has become indispensable in diagnosing CA [5]. This technique not only facilitates the distinction between ATTR and other forms of amyloidosis but also plays a crucial role in the early detection of cardiac deposits; consequently, its use has been included in international guidelines [1, [6]. Importantly, recent data have shown a higher prevalence of ATTR in specific cardiac patient subpopulations; however, given the inherited nature of ATTRv, genetic screening of asymptomatic family members is a vital strategy for managing this condition [1, 7–11]. The early identification of cardiac and neurological involvement permits the commencement of treatments such as TTR stabilisers or gene-silencing therapies, which may halt or decelerate the progression of the disease [12–16]. Therefore, early detection and precise assessment of cardiac involvement are imperative for enhancing patient outcomes, as therapeutic interventions are most efficacious when initiated prior to extensive cardiac deterioration. However, the is growing evidence on the results of pre-symptomatic scintigraphic screening in ATTR. This prospective, single-center study sought to evaluate the utility of pre-symptomatic scintigraphic imaging and genetic cascade screening for early detection of cardiac ATTR.

## Methods

### Study population and study protocol

**Fig. 1:**
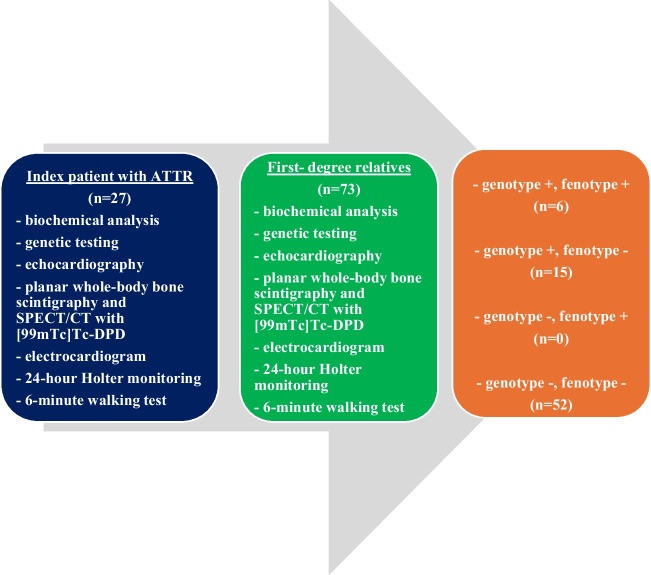
Study flowchart. ATTR- transthyretin amyloidosis; CT – computed tomography; DPD—3,3-disphono-1,2-propanodicarboxylic acid; SPECT—single-photon emission computed tomography

The study was conducted as multimodal and genetic cascade screening for relatives of individuals with ATTR supported by Pfizer Research Grant (ID#57165999) (ClinicalTrials.gov Identifier: NCT05814380). This prospective study took place at a tertiary cardiac centre from 2020 to 2024. It included 100 consecutive adults who met the enrolment criteria (Fig. 1). The analysis encompassed clinical data, biochemical analysis, free light chain blood immunoglobulins, urine immunofixation, electrocardiograms (ECG), 24-h Holter monitoring, transthoracic echocardiography (TTE), a 6-min walking test, planar whole-body bone scintigraphy and single-photon emission computed tomography/computed tomography (SPECT/CT) with Technetium 99 m and DPD tracer ([99mTc]Tc-DPD) irrespectively of predicted age of onset of disease (PADO). The ATTR cardiomyopathy diagnosis was established strictly in line with current guidelines and was characterized by the presence of grade 2 or 3 [99mTc]Tc-DPD uptake in planar whole-body scintigraphy, in the absence of monoclonal proteins in blood and urine [1, 4–6]. In selected cases, where the non-invasive algorithm provided unequivocal results, a cardiac or soft tissue biopsy was performed to confirm the final diagnosis [4–6]. Patients underwent genetic testing using an amplicon-based next-generation TTR sequencing approach. Those with positive free light chain blood immunoglobulins or urine immunofixation were referred to a haematology specialist and underwent a bone marrow biopsy. The inclusion criteria required participants to be over 18 years of age, provide written informed consent, and be diagnosed with ATTR or be a first-degree relative of a patient with ATTR. Exclusion criteria included pregnancy or any other previously diagnosed pre-existing infiltrative disorders. The study population was divided into the following four groups: (1) index patients with ATTR CA, (2) relatives with ATTR CA, (3) ATTRv carriers without ATTR CA, and (4) genotype and phenotype negative relatives.

### Echocardiography

Echocardiographic examinations were conducted using a Philips EPIQ7 device (The Netherlands) by operators experienced and blinded to the eventual diagnoses, consistent with prevailing guidelines [5, 17]. The assessment included standard views for planar measurements, M-mode, and Doppler modalities such as continuous, pulsed, tissue, and Colour Doppler. Longitudinal left ventricular (LV) strain curves were manually derived from the apical 2-, 3-, and 4-chamber views. The global LV longitudinal strain (GLS), calculated from the peak negative instantaneous average across 18 longitudinal segmental strains, was also evaluated [18]. Furthermore, we have conducted a comprehensive assessment of the applicability and performance of two previously published ATTR cardiac amyloidosis risk scores, the ATTR-CM score and the IWT (index of wall thickness) score, within our patient cohort [19, 20]. This analysis aimed to evaluate the utility and limitations of these diagnostic tools in identifying ATTR CA, particularly in the early, asymptomatic stages of the disease.

### [99mTc]Tc-DPD planar whole-body bone scintigraphy and SPECT/CT

The procedure and image acquisition of [99mTc]Tc-DPD adhered to the current recommendations [5]. Briefly, Technetium 99 m (370–740 MBq) and DPD tracer (TECEOS, CIS BIO) were applied. The imaging protocol included planar whole-body bone scans 2–3 h following intravenous [99mTc]Tc-DPD administration. The planar scintigraphic scans were assessed according to the Perugini semi-quantitative scale (grade 0 (no myocardial uptake and normal bone uptake), grade 1 (myocardial uptake less than rib uptake), grade 2 (myocardial uptake equal to rib uptake), and grade 3 (myocardial uptake greater than rib uptake with mild/absent rib uptake)) [5, 21]. Furthermore, computed tomography (CT) attenuation-corrected and non-corrected single photon emission tomography (SPECT) images were assessed in the coronal, transaxial, and sagittal planes, as well as in tridimensional maximal-intensity projection cine mode by three experienced nuclear medicine specialists, blinded to final diagnosis. Bone scintigraphy served as the gold standard diagnostic tool for ATTR CA, while SPECT/CT was primarily used to identify and rule out false-positive radiotracer uptake.

### Statistical analysis

For quantitative variables, ANOVA was used if the assumptions of homogeneity of variance (Levene's Test) and normality of residuals (QQ plot) were met. If these assumptions were not met, a Box-Cox transformation was applied, and the assumptions were checked again. If the assumptions were met after transformation, ANOVA was conducted on the transformed data. In case the assumptions were still not met, the non-parametric Kruskal–Wallis ANOVA was used. If significant differences were found between groups, a post hoc analysis was performed. Qualitative variables were analysed using the chi-square test of independence. If a dependency was found, pairwise comparisons were performed with a Bonferroni correction for multiple testing. P-values less than 0.05 were considered statistically significant. Statistical analyses were conducted using Statistica 13.0 and MedCalc software. Data underlying this article will be made available upon a reasonable request to the corresponding author.

### Ethical standards

Written informed consent was obtained from all participants enrolled in the study. All procedures were conducted in compliance with the ethical standards set forth by the local Ethics Committee (103/KBL/OIL/2020), the 1964 Helsinki Declaration, and its subsequent amendments, or comparable ethical standards.

## Results

**Fig. 2:**
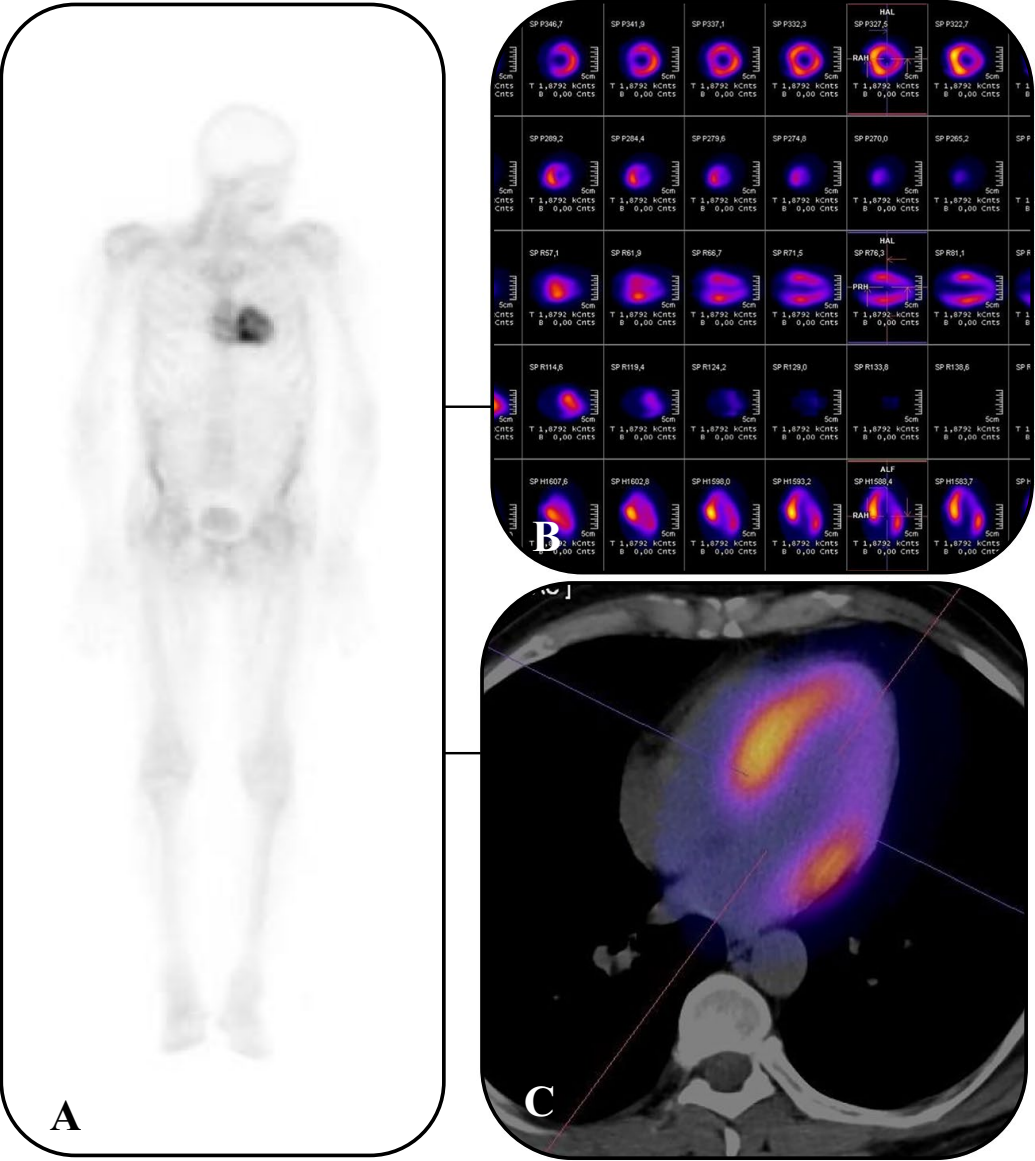
Imaging results in an asymptomatic first-degree relative of a patient with hereditary cardiac transthyretin amyloidosis are consistent with advanced cardiac involvement. A—planar whole-body scintigraphy with [99mTc] Tc-DPD (grade 3). B—SPECT imaging with [99mTc]Tc-DPD (after attenuation correction). C – hybrid SPECT/CT imaging. CT – computed tomography; DPD—3,3-disphono-1,2-propanodicarboxylic acid; SPECT—single-photon emission computed tomography

**Fig. 3:**
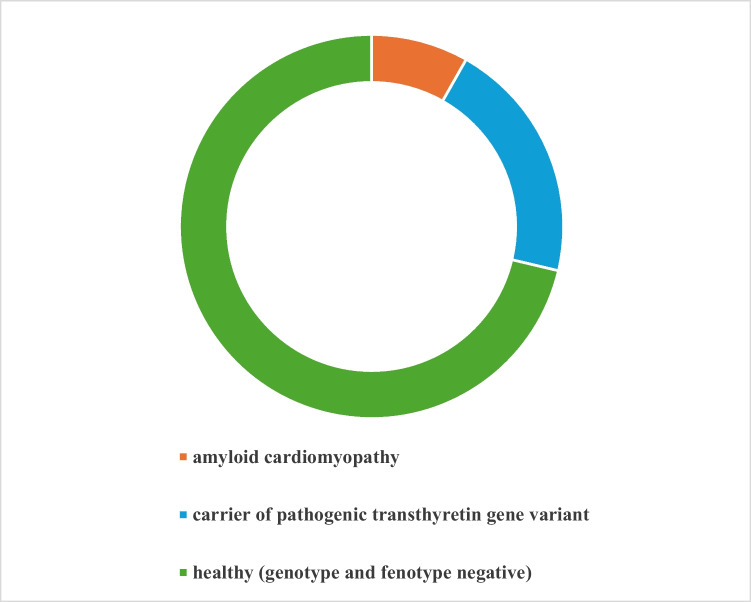
Final diagnosis within asymptomatic first-degree relatives of patients with transthyretin amyloidosis

Between 2020 and 2024, a total of 100 consecutive patients were enrolled in the study, comprising 27 index patients (41% ATTRv and 59% ATTRwt) and 73 asymptomatic first-degree relatives (Fig. 2). Within the group of 73 first-degree relatives, 6 (8.2%) presented with findings consistent with ATTRv CA, namely radiotracer uptake in [99mTc]Tc-DPD scintigraphy and a pathogenic TTR variant (Fig. 3). Moreover, 15 of first-degree relatives (20.5%) had a pathogenic TTR variant but exhibited no radiotracer uptake in [99mTc]Tc-DPD scintigraphy (time to PADO ≤ 29 years). Importantly, none of the relatives of ATTRwt patients displayed ATTR CA in [99mTc]Tc-DPD scintigraphy or carried a pathogenic TTR variant.

The study population was divided into the following four groups: (1) index patients with ATTR CA (G ± P +), (2) relatives with ATTR CA (G + P +), (3) ATTRv carriers without ATTR CA (G + P-), and (4) genotype and phenotype negative relatives (G-P-). The baseline demographic, clinical, and laboratory characteristics of the study populations are presented in Table 1. The presence of TTR variants was significantly different across the groups, with 41% in index patients with ATTR CA (8 Phe53Leu, 1 Glu109Lys, 1 Glu122Lys, 1 Glu82Lys), 100% in relatives with ATTR CA (6 Phe53Leu), 100% in ATTRv carriers without ATTR CA (14 Phe53Leu, 1 Glu122Lys), and 0% in genotype and phenotype negative relatives (*p* < 0.05). Overall, among identified TTR variants, there was a predominance of one pathogenic variant c.157 T > C p. (Phe53Leu) (87.5%).

**Table 1:** Baseline demographic, clinical and laboratory characteristics of the study population, which was divided into the following groups: index patients with ATTR CA (G ± P +), relatives with ATTR CA (G + P +), ATTRv carriers without ATTR CA (G + P-), genotype and phenotype negative relatives (G-P-)

Variable	Index patients with ATTR CA* (*n* = 27)	Relatives with ATTR CA* (*n* = 6)	ATTRv carriers without ATTR CA* (*n* = 15)	Genotype and phenotype negative relatives* (*n* = 52)	*p* value
Female gender	**5 (18%)**	2 (33%)	**9 (60%)**	**36 (69%)**	** < 0.05**
Body mass index (kg/m2)	26.9 ± 4.6	26.8 ± 3.9	24.5 ± 4.5	24.6 ± 4.5	0.29
Age (years)	**69.7 ± 11.2**	57.5 ± 9.8	**41.3 ± 13.6**	**45.5 ± 12.0**	** < 0.001**
Amyloidosis type -ATTRv -ATTRwt	**- 11 (41%)** **- 16 (59%)**	**- 6 (100%)** **-0 (0%)**	**- 15 (100%)** **-0 (0%)**	**-0 (0%)** **-0 (0%)**	** < 0.05**
Transthyretin variants present	**11 (41%)**	**6 (100%)**	**15 (100%)**	**0 (0%)**	** < 0.001**
Chronic kidney disease	**15 (55%)**	0 (0%)	**0 (0%)**	**1 (2%)**	** < 0.001**
Bicep tendon rupture	2 (7%)	0 (0%)	1 (7%)	0 (0%)	0.23
Lumbar spine stenosis	6 (22%)	1 (17%)	2 (13%)	8 (16%)	0.86
Cardiac implantable electronic device present	**12 (44%)**	1 (17%)	**0 (0%)**	**0 (0%)**	** < 0.001**
Diabetes	**10 (37%)**	0 (0%)	**0 (0%)**	**2 (4%)**	** < 0.001**
Arterial hypertension	10 (37%)	1 (17%)	3 (20%)	14 (27%)	0.06
Coronary artery disease	**17 (63%)**	2 (33%)	**0 (0%)**	**0 (0%)**	** < 0.001**
Weight loss over the previous 6 months	**4 (15%)**	0 (0%)	0 (0%)	**0 (0%)**	**0.009**
NYHA III-IV class	**14 (52%)**	0 (0%)	**0 (0%)**	**0 (0%)**	** < 0.001**
Systolic blood pressure (mmHg)	**112.6 ± 18.3**	123.3 ± 7.7	116.5 ± 16.5	**128.3 ± 20.0**	**0.005**
Heart rate (beats per minute)	71.4 ± 11.9	77.5 ± 13.1	70.0 ± 13.1	73.2 ± 16.3	0.69
Pulmonary congestion	**7 (26%)**	0 (0%)	0 (0%)	**0 (0%)**	** < 0.001**
Haematocrit (%)	40.2 ± 4.5	43.4 ± 2.6	40.8 ± 4.6	41.0 ± 4.1	0.42
INR	**1.3 ± 0.7**	1.0 ± 0.1	**0.9 ± 0.1**	**1.0 ± 0.1**	** < 0.001**
Creatinine (mg/dl)	**111.9 ± 39.8**	74.2 ± 14.5	**66.5 ± 14.7**	**68.8 ± 14.5**	** < 0.001**
eGFR (ml/min/1,73m2)	**63.7 ± 22.4**	90.5 ± 16.3	**113.0 ± 12.9**	**100.4 ± 16.6**	** < 0.001**
Urea (mmol/l)	**10.1 ± 5.7**	5.0 ± 0.9	**4.1 ± 1.3**	**4.9 ± 2.0**	**0.006**
Aspartate transaminase (U/L)	**45.0 ± 98.2**	26.2 ± 7.8	**19.8 ± 5.0**	23.8 ± 9.4	**0.03**
Bilirubin (μmol/L)	**22.5 ± 20.5**	12.2 ± 8.9	**9.2 ± 4.6**	**10.6 ± 10.8**	** < 0.001**
GGTP (U/L)	**85.9 ± 84.1**	105.5 ± 158.6	**24.6 ± 17.9**	**29.2 ± 26.2**	** < 0.001**
ALP (U/L)	**109.7 ± 58.1**	121.0 ± 61.6	**70.6 ± 23.7**	**76.7 ± 23.2**	**0.006**
NT-proBNP (pg/mL)	**3165.0 [1581; 8385]**	164.5 [79.0; 1235.0]	**50.5 [23.0; 74.0]**	**56.0 [34.0; 80.0]**	** < 0.001**
Cardiac troponin T (ng/mL)	**0.07 [0.040; 0.097]**	**0.02 [0.011; 0.023]**	**0.003 [0.003; 0.004]**	**0.004 [0.003; 0.005]**	** < 0.001**
Albumin (g/L)	**46.219 ± 52.3**	41.4 ± 2.3	39.3 ± 2.7	**39.3 ± 3.1**	**0.007**
Uric Acid (μmol/L)	**441.9 ± 154.1**	298.8 ± 30.3	**271.9 ± 60.6**	**274.7 ± 62.9**	** < 0.001**
ECG—low voltage	**16 (59%)**	1 (17%)	**3 (20%)**	**3 (6%)**	** < 0.001**
ECG – pathological q	**2 (7%)**	**3 (50%)**	**0 (0%)**	**1 (2%)**	** < 0.001**
ECG –ST-T abnormalities	**12 (44%)**	1 (17%)	2 (13%)	**5 (10%)**	** < 0.001**
Atrioventricular block	**12 (44%)**	2 (33%)	**0 (0%)**	**0 (0%)**	** < 0.001**
6-min walking test distance (m)	**277.1 ± 159.9**	426.5 ± 151.5	**486.6 ± 50.6**	**505.2 ± 75.9**	** < 0.001**

^*^ The data is given as a number (percentage) for categorical data, and as a mean value ± one standard deviation or median (IQR) for continuous variables. Values considered statistically significant are indicated in bold font. AL—light chain amyloidosis; ATTR- transthyretin amyloidosis; ATTRwt—transthyretin amyloidosis wild-type; ATTRv—hereditary transthyretin amyloidosis; CA—cardiac amyloidosis; ECG – electrocardiogram; NYHA—New York Heart Association class; NT-proBNP—N-terminal pro-brain natriuretic peptide

In the group of index patients with ATTR CA, there was a predominance of male participants, and the mean age was significantly higher compared to ATTRv carriers without ATTR CA and genotype- and phenotype-negative relatives (*p* < 0.05). Additionally, chronic kidney disease, coronary artery disease, cardiac implantable electronic devices, and diabetes were significantly more prevalent in this population (*p* < 0.001 for all). Index patients with ATTR CA presented with a higher NYHA functional class and elevated levels of high-sensitivity troponin and N-terminal pro-brain natriuretic peptide (NT-proBNP) when compared to G + P- and G-P- groups (*p* < 0.001). Moreover, index patients with ATTR CA exhibited a higher incidence of low-voltage electrocardiographic findings, pathological Q waves, and a greater burden of atrioventricular block on Holter monitoring compared to ATTRv carriers without ATTR CA and genotype and phenotype negative relatives (*p* < 0.001). Finally, the 6-min walking test distance was significantly shorter in the group of index patients with ATTR CA (*p* < 0.001). Relatives with ATTR CA presented with an elevated levels of high-sensitivity troponin, and exhibited a higher incidence of pathological Q waves when compared to G + P- and G-P- groups (*p* < 0.001).

The distribution of echocardiographic findings within the groups are presented in Table 2. Overall, echocardiographic evaluation revealed greater values of LV maximum wall thickness, LV mass index, left atrium area and volume, reduced LV ejection fraction (LVEF), GLS, as well as more advanced diastolic dysfunction in index patients with ATTR CA compared to ATTRv carriers without ATTR CA and genotype and phenotype negative relatives (*p* < 0.001). These findings were accompanied by a notable decline in tissue velocities measured in Tissue Doppler Imaging (TDI) in this group (*p* < 0.001). Moreover, index patients with ATTR CA exhibited a significantly greater thickness of the right ventricle (RV) wall, as well as increased dimensions of the right atrium area and volume compared to G + P- and G-P- groups (*p* < 0.001). Moreover, these findings were followed by a significant decline in RV systolic function [TDI RV S’, tricuspid annular plane systolic excursion (TAPSE), and RV outflow tract velocity time integral (VTI); *p* < 0.05 for all]. Using echocardiography, it was found that index patients with ATTR CA more frequently presented with distinct characteristics such as ‘apical sparing’ and the presence of the ''5–5-5'' sign, wherein tissue velocities {‘s’ [systolic], e’ [early diastolic], and a’ [late (atrial) diastolic]} measured in TDI were all below 5 cm/s (*p* < 0.001).

**Table 2:** Echocardiographic findings within the following groups: index patients with ATTR CA (G ± P +), relatives with ATTR CA (G + P +), ATTRv carriers without ATTR CA (G + P-), genotype and phenotype negative relatives (G-P-)

Variable	Index patients with ATTR CA* (*n* = 27)	Relatives with ATTR CA* (*n* = 6)	ATTRv carriers without ATTR CA* (*n* = 15)	Genotype and phenotype negative relatives* (*n* = 52)	*p* value
Left ventricle intraventricular septum thickness (mm)	**20.2 ± 2.6**	**15.7 ± 3.9**	**10.2 ± 1.7**	**11.1 ± 2.3**	** < 0.001**
Left ventricle posterior wall thickness (mm)	**18.5 ± 3.9**	**15.6 ± 3.8**	**9.8 ± 1.9**	**10.6 ± 2.1**	** < 0.001**
Left ventricle maximum wall thickness (mm)	**21.8 ± 2.9**	**15.7 ± 1.9**	**10.5 ± 1.7**	**11.7 ± 2.2**	** < 0.001**
LV mass index (g/m2)	**219.9 ± 54.0**	160.7 ± 64.2	**84.9 ± 21.7**	**96.3 ± 26.5**	** < 0.001**
Relative wall thickness (RWT—sum of septal and posterior wall thickness divided by left ventricular end diastolic diameter)	**0.92** ± 0.23	0.74 ± 0.27	**0.45** ± 0.1	**0.48** ± 0.1	** < 0.001**
Left ventricle end-diastolic diameter (mm)	43.7 ± 7.1	44.2 ± 7.6	45.1 ± 4.8	45.4 ± 5.7	0.71
Left ventricle end-diastolic volume (ml)	101.1 ± 43.3	93.7 ± 39.1	101.4 ± 19.8	96.1 ± 27.0	0.75
Left atrium area (cm^2^)	**31.2 ± 6.3**	21.6 ± 2.0	**17.6 ± 4.2**	**17.2 ± 3.9**	** < 0.001**
Left atrium volume index (ml/m^2^)	**57.7 ± 19.5**	39.8 ± 12.3	**26.6 ± 9.0**	**25.7 ± 9.4**	** < 0.001**
Left ventricular ejection fraction (%)	**40.6 ± 12.5**	55.8 ± 9.3	**61.6 ± 4.0**	**62.3 ± 4.9**	** < 0.001**
Stroke volume (ml)	54.0 ± 20.8	70.7 ± 18.7	67.1 ± 0.7	61.7 ± 20.5	0.14
Left ventricular VTI (cm)	**17.1 ± 6.1**	24.6 ± 5.2	22.4 ± 4.2	**21.7 ± 4.8**	**0.003**
Global longitudinal strain (GLS, -%)	**12.7 ± 6.1**	14.7 ± 3.7	**19.4 ± 3.5**	**19.2 ± 3.6**	** < 0.001**
Septal apical to base longitudinal strain ratio (SAB)	11.6 ± 34.8	31.0 ± 69.1	24.7 ± 86.1	2.3 ± 2.4	0.17
E/A	**2.1 ± 0.9**	**1.1 ± 0.4**	1.4 ± 0.4	**1.2 ± 0.4**	**0.005**
E/E’	**20.1 ± 9.7**	15.2 ± 11.8	**6.1 ± 1.9**	**7.3 ± 1.8**	** < 0.001**
Left ventricle lateral wall TDI S’(cm/s)	**4.5 ± 1.1**	6.2 ± 2.3	**9.0 ± 2.3**	**8.1 ± 2.5**	** < 0.001**
Left ventricle lateral wall TDI E’ (cm/s)	**6.4 ± 2.5**	**8.4 ± 3.2**	**15.2 ± 3.5**	**13.7 ± 4.5**	** < 0.001**
Left ventricle lateral wall TDI A’ (cm/s)	**4.5 ± 2.3**	8.9 ± 5.2	**9.4 ± 2.6**	**9.9 ± 2.9**	** < 0.001**
Left ventricle intraventricular septum TDI S’ (cm/s)	**4.2 ± 1.4**	5.3 ± 1.6	**7.2 ± 1.2**	**7.3 ± 1.6**	** < 0.001**
Left ventricle intraventricular septum TDI E’ (cm/s)	**6.6 ± 12.3**	**5.2 ± 1.9**	**10.2 ± 2.0**	**10.7 ± 7.9**	** < 0.001**
Left ventricle intraventricular septum TDI A’ (cm/s)	**4.1 ± 2.7**	**8.4 ± 4.4**	**8.2 ± 2.1**	**8.6 ± 2.6**	** < 0.001**
Right ventricle thickness (mm)	**7.2 ± 2.3**	6.5 ± 2.8	**4.3 ± 1.1**	**4.7 ± 1.9**	** < 0.001**
Right ventricle outflow tract diameter (mm)	**34.8 ± 7.0**	30.2 ± 3.9	**28.6 ± 5.0**	**27.9 ± 4.7**	** < 0.001**
Right atrium area (cm^2^)	**27.4 ± 7.3**	19.1 ± 4.8	**15.0 ± 3.5**	**14.6 ± 3.9**	** < 0.001**
Right atrium volume index (ml/m^2^)	**50.3 ± 16.1**	34.2 ± 15.7	**19.8 ± 5.7**	**20.3 ± 6.6**	** < 0.001**
Right ventricle VTI (cm)	**13.5 ± 5.4**	17.6 ± 2.9	**16.3 ± 3.4**	**17.6 ± 3.8**	**0.003**
TAPSE (mm)	**14.8 ± 5.4**	19.2 ± 4.7	**23.9 ± 3.5**	**22.8 ± 3.2**	** < 0.001**
sPAP (mmHg)	**37.0 ± 9.7**	**18.8 ± 9.2**	**21.4 ± 5.8**	**22.0 ± 5.0**	** < 0.001**
Right ventricle TDI S’ (cm/s)	**8.3 ± 3.0**	9.9 ± 3.3	**12.3 ± 2.1**	**12.5 ± 2.3**	** < 0.001**
Right ventricle TDI E’ (cm/s)	**8.1 ± 3.3**	8.8 ± 3.4	**11.7 ± 4.4**	**12.9 ± 3.6**	** < 0.001**
Right ventricle TDI A’(cm/s)	**7.7 ± 6.2**	9.7 ± 3.6	**13.8 ± 5.7**	**13.3 ± 3.9**	** < 0.001**
Apical sparing	**21 (78%)**	4 (67%)	**3 (20%)**	**16 (31%)**	** < 0.001**
Ground glass appearance of myocardium	25 (93%)	6 (100%)	13 (87%)	38 (73%)	0.89
''5–5-5'' sign (s' [systolic], e' [early diastolic], and a' [late (atrial) diastolic] tissue velocities are all < 5 cm/s)	**11 (41%)**	**1 (17%)**	**0 (0%)**	**0 (0%)**	** < 0.001**
Pericardial effusion (mm)	**5.7 ± 5.4**	0.8 ± 1.3	**0.5 ± 1.3**	**0.3 ± 0.7**	** < 0.001**

^*^ The data is given as a number (percentage) for categorical data, and as a mean value ± one standard deviation or median (IQR) for continuous variables. Values considered statistically significant are indicated in bold font. ATTR- transthyretin amyloidosis; ATTRwt—transthyretin amyloidosis wild-type; ATTRv—hereditary transthyretin amyloidosis; CA—cardiac amyloidosis; cm – centimeter; LV – left ventricle; mmHg—millimeters of mercury; ml – milliliter; mm – millimeter; TAPSE—tricuspid annular plane systolic excursion; TDI –Tissue Doppler Imaging; s – second; sPAP—systolic pulmonary artery pressure; VTI -Velocity Time Integral

Moreover, relatives with ATTR CA exhibited significantly greater thickness of the intraventricular septum and left ventricular posterior wall compared to ATTRv carriers and genotype and phenotype negative relatives (*p* < 0.001). These individuals also demonstrated a notable decline in lateral wall and intraventricular septal E' tissue velocities measured by TDI, relative to G + P- and G-P- groups (*p* < 0.001). Furthermore, compared to index patients with ATTR CA, the relatives with ATTR CA presented with lower E/A and higher intraventricular septal A' tissue velocities, as measured by TDI (*p* < 0.01). Echocardiographic assessment revealed that relatives with ATTR CA more frequently displayed the presence of the "5–5-5" sign compared to genotype and phenotype negative relatives, and higher systolic pulmonary artery pressure compared to index patients with ATTR CA (*p* < 0.001).

Table 3 summarises the scintigraphic findings across the four study groups. In index patients with ATTR CA and relatives with ATTR CA, all participants exhibited [99mTc]Tc-DPD radiotracer uptake in the cardiac region. In contrast, no such radiotracer accumulation was observed in ATTRv carriers without ATTR CA (G + P-) and genotype and phenotype negative relatives (G-P-) (*p* < 0.001). Notably, in SPECT/CT there was an absence of tracer uptake in the apical segments in 83% asymptomatic subjects with positive scintigraphy, consistent with an apical sparing pattern. This finding suggests that apical sparing may serve as an early indicator of CA, particularly in patients who remain asymptomatic.

**Table 3:** Scintigraphic assessment within the following groups: index patients with ATTR CA (G ± P +), relatives with ATTR CA (G + P +), ATTRv carriers without ATTR CA (G + P-), genotype and phenotype negative relatives (G-P-)

Variable	Index patients with ATTR CA* (*n* = 27)	Relatives with ATTR CA* (*n* = 6)	ATTRv carriers without ATTR CA* (*n* = 15)	Genotype and phenotype negative relatives* (*n* = 52)	*p* value
Present [99mTc]Tc- DPD tracer uptake in cardiac region	**27 (100%)**	**6 (100%)**	**0 (0%)**	**0 (0%)**	** < 0.001**
Perugini semi-quantitative grade - 0 - 1 - 2 - 3	**0 (0%)** **1 (4%)** **5 (18%)** **21 (78%)**	**0 (0%)** **0 (0%)** **1 (17%)** **5 (83%)**	**15 (100%)** **0 (0%)** **0 (0%)** **0 (0%)**	**52 (100%)** **0 (0%)** **0 (0%)** **0 (0%)**	** < 0.001**

^*^ The data is given as a number (percentage) for categorical data, and as a mean value ± one standard deviation or median (IQR) for continuous variables. Values considered statistically significant are indicated in bold font. ATTR- transthyretin amyloidosis; CA—cardiac amyloidosis

The high-risk (≥ 6) ATTR-CM risk score demonstrated diagnostic performance, with a sensitivity of 81.82% and a specificity of 91.04% in the general population. The positive predictive value was 81.82%, and the negative predictive value was 91.04%, leading to an overall accuracy of 88.00%. However, when the population was divided into index patients with ATTR CA and their relatives, the sensitivity was significantly higher in the index patients (88.89%) compared to the relatives (50.00%), with a p-value of 0.0278. The optimal cut-off points for maximizing the Youden index was identified as 3.5, which resulted in a sensitivity of 96.97% and a specificity of 88.06%. In contrast, the high-risk IWT score (≥ 8) exhibited a lower sensitivity of 30.30%, despite a high specificity of 100.00%. The positive predictive value was 100.00%, but the negative predictive value was lower at 74.44%, yielding an overall accuracy of 77.00%. The differences in sensitivity between the index patients and their relatives were not statistically significant, with a sensitivity of 33.33% in the index patients versus 16.67% in the relatives (*p* = 0.429). When the cut-off point was adjusted to 3.5, the sensitivity of the IWT score improved to 84.85%, and the specificity was 95.52%, resulting in an overall accuracy of 92.00%.

## Discussion

Presymptomatic genetic testing is currently recommended for individuals with a family history of ATTRv [1, 22, 23]. This enables the identification of carriers of TTR gene variants, facilitating timely interventions and regular follow-ups to detect the earliest signs of the disease. Firstly, our data analysis shows that 8.2% of asymptomatic family members of patients with ATTR may have undiagnosed CA. Importantly, according to presented results, routine screening in families with ATTRwt is not justified.

The TTR variants associated with ATTR amyloidosis exhibit significant variability in their genotypic and phenotypic characteristics across different geographical regions and ethnic populations [24–27]. Globally, some of the most common TTR variants include Val122Ile, which affects approximately 3.4% of African Americans, while in the European THAOS registry, the Val30Met variant is the predominant pathogenic variant, particularly prevalent in Portugal; this is followed by the Ile68Leu variant, which is endemic to Italy [28, 29]. In the present study, we identified several rare types of ATTR variants, affecting 28.7% of first-degree relatives, with the Phe53Leu variant being the most frequent. Conversely, the prevalence of the V122I TTR variant reaches 3.2% in participants of the Dallas Heart Study [30]. Importantly, V122I TTR carriers were at a higher adjusted risk of heart failure (HR 3.82, 95% CI 1.80–8.13, *P* < 0.001), cardiovascular death (HR 2.65, 95% CI 1.14–6.15, *P* = 0.023), and all-cause mortality (HR 1.95, 95% CI 1.08–3.51, *P* = 0.026) compared to non-carriers. Furthermore, in a large population-based cohort with exome sequencing data, participants with pathogenic or likely pathogenic TTR variants, exhibited a significantly increased odds of heart disease (including heart failure, cardiomyopathy, and atrial fibrillation) [31].

Current guidelines emphasise the importance of establishing a baseline for crucial clinical markers and determining the schedule of follow-up assessments based on PADO [32]. Monitoring should begin 10 years before the PADO, with annual check-ups increasing in frequency as individuals approach their PADO, particularly for genotypes linked to rapid progression [32]. In our study cohort, patients underwent comprehensive cascade screening regardless of their time to PADO. Notably, all relatives diagnosed with ATTR CA had up to nine years until their PADO. This observation aligns with and reinforces previous recommendation [32].

In recent years, nuclear imaging techniques have been gradually developing towards wider clinical application in the diagnosis of ATTR [1, 4–6, 33–35]. Scintigraphic imaging using bone-targeting radiotracers provides a non-invasive approach to detect amyloid deposits in the heart and other tissues [1, 4–6, 33–35]. The high sensitivity of scintigraphy in identifying early-stage amyloid deposition can enable timely therapeutic interventions, which may potentially slow disease progression and improve the quality of life for affected individuals. However, it is important to note that current recommendations do not specify the appropriate patient populations or the timing for performing scintigraphic assessments [32]. Furthermore, advancements in imaging technology have led to the development of SPECT/CT, which offers enhanced spatial resolution and improved diagnostic, and prognostic capabilities compared to planar imaging [36–43]. While planar scintigraphy remains the gold standard and guideline-recommended imaging technique for diagnosing ATTR CA, this study incorporated SPECT/CT as well due to its superior spatial resolution and ability to quantify the degree and pattern of myocardial radiotracer accumulation [4–6]. As SPECT/CT modality continues to evolve, it holds promise not only for enhancing diagnostic precision but also for contributing to the higher sensitivity in high populations based on more detailed assessment of disease burden and distribution. The absence of tracer uptake in the apical segments, as observed in 83% of our cohort of asymptomatic subjects, highlights the importance of recognising apical sparing as a potential marker of early CA. This pattern, which mirrors findings in echocardiography and is associated with mortality, could aid in the early identification of the disease before the onset of symptoms, potentially improving patient outcomes [38].

The presented findings demonstrate that the implementation of a systematic screening protocol integrating both genetic and scintigraphic assessments can significantly improve the early identification of ATTR amyloidosis. This comprehensive approach can be particularly beneficial for populations at risk, enabling healthcare professionals to develop personalised monitoring and treatment strategies. The recent study examined the effectiveness of the 2021 European Society of Cardiology (ESC) screening recommendations for individuals with a pathogenic TTR variant [44]. The study included 159 at-risk relatives from 10 centres, with a primary endpoint of cardiac tracer uptake or a transthyretin-positive cardiac biopsy. Importantly, 25% of these relatives were diagnosed with ATTRv CA at baseline, with half meeting the secondary endpoint of heart failure or pacemaker-requiring conduction disorders. Importantly, 13% of relatives with ATTRv CA did not show any signs of cardiac involvement on first-line tests, highlighting the value of scintigraphy for early ATTR detection. The study also demonstrated the utility of serial evaluation, with a 9.4% yield at a 3-year follow-up, supporting the ESC's recommendation for periodic reassessment. While consistent with these findings, our study introduces significant novelty by adopting a prospective screening approach regardless of the time to PADO. This allows us to capture a more comprehensive understanding of the natural history and progression of ATTR in at-risk individuals, regardless of their current disease stage. Additionally, our study uniquely includes family members of patients with ATTRwt, a population not addressed in the previous study.

There are data suggesting that implementation of ATTR CA screening protocols may be beneficial. The retrospective study evaluated the impact of implementing a structured CA clinical pathway on patient outcomes [45]. The study compared patient data from two periods: pre-implementation (2007–2018) and post-implementation (2019–2020) of the clinical pathway. Following the implementation, there was a significant reduction in the diagnostic delay, decreasing from 14 to 8 months. Additionally, the severity of disease at diagnosis was reduced, with the proportion of patients presenting with advanced CA (Mayo/Gillmore Stage III/IV) dropping from 61 to 33%. The study also noted increased awareness among cardiologists. Moreover, a multicentre retrospective study involving 1,281 patients diagnosed with ATTRwt CA across 17 Italian referral centres categorised patients into different diagnostic pathways: hypertrophic cardiomyopathy (HCM) pathway (7%), heart failure (HF) pathway (51%), incidental imaging (23%), and incidental clinical findings (19%) [46]. Notably, patients diagnosed via the HF pathway were older, with a mean age of 78 years, and had a worse clinical profile, including higher NYHA class III-IV and a greater prevalence of chronic kidney disease. Importantly, these patients also exhibited poorer survival outcomes compared to those diagnosed through the other pathways. The study highlighted the importance of identifying and understanding the various diagnostic triggers, particularly in patients presenting with HF. Achten et al. provided insights into the diagnostic trajectory of ATTR CA over a six-year period, involving 65 patients, predominantly with ATTRwt CA (91%) and a minority with ATTRv CA (9%) [47]. Despite increased awareness and the adoption of less invasive diagnostic techniques, the study observed no significant reduction in the time from HF symptom onset to diagnosis, with a median duration of approximately 27 to 30 months. This delay was primarily driven by prolonged time to referral, even in the presence of red flags. The study underscores the persistent challenges in achieving timely diagnosis, despite the presence of typical clinical indicators. While those studies demonstrated the benefits of a structured clinical pathway in reducing diagnostic delays and disease severity at diagnosis, our study also highlights the importance of implementing a systematic screening. However, our study provides a broader assessment by including both ATTRwt and ATTRv, and by incorporating asymptomatic family members, which offers a more comprehensive understanding of the disease spectrum. Additionally, our findings suggest that systematic prospective screening, especially among asymptomatic relatives, could potentially mitigate the diagnostic delays observed, ultimately leading to earlier intervention and better patient outcomes.

Importantly, echocardiography is a key technique for monitoring patients with CA, but it may have diagnostic limitations in certain subpopulations, such as those with ATTRwt, as the increased ventricular wall thickness is not always very prominent [48]. In our study, the ATTR-CM risk score demonstrated superior overall diagnostic accuracy, particularly when using an adjusted cut-off value [19]. However, it displayed significantly lower sensitivity (50%) in the relatives of ATTR patients who were diagnosed with ATTR CA. In contrast, the IWT score exhibited high specificity, but notably lower sensitivity, especially in the preclinical stage of the disease (16.67%), limiting its effectiveness as an early diagnostic tool [20]. These findings indicate that the current echocardiographic risk assessment tools have significant limitations in reliably identifying individuals with ATTRv CA during the early, asymptomatic stages of the disease. Based on the observations from this study, these traditional echocardiographic scores were unable to diagnose more than half of the patients who, at that point, already exhibited positive scintigraphic evidence and confirmed ATTR CA. This underscores the need for a more comprehensive, multi-modal approach to screening and detection, incorporating advanced imaging techniques like bone scintigraphy, in order to enhance the early identification of this condition, especially in at-risk populations. This analysis supports the conclusion that echocardiographic screening alone may not be sufficient, and that diagnostic scintigraphy should remain a cornerstone of early ATTR detection, especially in high-risk populations such as first-degree relatives of ATTRv patients. These results underscore the need for further research and refinement of ATTRv CA risk assessment tools to improve their ability to accurately identify individuals with early-stage or preclinical ATTR CA, particularly among first-degree relatives of ATTR patients.

## Limitations

As this was a single-centre study, the findings require validation in a multi-centre setting. Furthermore, all participants were of Caucasian ethnicity due to the local demographics, which may limit the generalisability of the results to more diverse geographical regions. This investigation was conducted at a cardiology referral centre specialising in heart failure and cardiomyopathies. Consequently, this may have introduced selection bias due to the referral of pre-selected patients. Additionally, the protocol primarily relied on non-invasive approaches and scintigraphic evaluation, with invasive soft tissue and/or endomyocardial biopsy performed only in patients with unequivocal results from the non-invasive algorithm [4]. While this methodological approach has inherent limitations, it has been adopted by leading experts in the field [4–6].

## Conclusions

The presented findings confirm that the implementation of a systematic screening protocol combining genetic, echocardiographic, and scintigraphic assessments can enhance the early identification of ATTR. Our study highlights that a significant proportion of asymptomatic relatives of patients with ATTRv may have underlying CA. In recent years, scintigraphic imaging has gained increasing recognition as a crucial non-invasive diagnostic tool for ATTR CA. Consequently, bone scintigraphy should be incorporated into the recommendations for the evaluation of ATTRv family members, alongside genetic testing, and echocardiographic examination, particularly for individuals approaching the age at which the typical phenotypic expression of their specific TTR variant is observed. However, based on our findings, relatives of patients with ATTRwt do not require more than standard cardiac investigations, as none were found to have ATTR cardiac amyloidosis in our comprehensive assessment. The findings demonstrate that the existing risk assessment instruments are not consistently effective in identifying individuals with ATTRv CA during the early, preclinical phases of the condition. This analysis supports the conclusion that echocardiographic screening alone may be inadequate, and that bone scintigraphy should continue to be a fundamental component of early detection, especially among high-risk populations such as first-degree relatives of ATTR patients. Further research is warranted to determine the long-term clinical implications and optimal management strategies for these pre-symptomatic individuals.

## Data Availability

Data underlying this article will be made available upon a reasonable request to the corresponding author.

## Abbreviations

ATTRwt: Transthyretin amyloidosis wild-type
ATTRv: Hereditary transthyretin amyloidosis
CA: Cardiac amyloidosis
CT: Computed tomography
DPD: 3,3-Disphono-1,2-propanodicarboxylic acid
PADO: Predicted age of onset of disease
SPECT: Single-photon emission computed tomography
TTE: Transthoracic echocardiography
TTR: Transthyretin
